# Simulation of Picking Up Metal Microcomponents Based on Electrochemistry

**DOI:** 10.3390/mi11010033

**Published:** 2019-12-26

**Authors:** Dongjie Li, Jiyong Xu, Weibin Rong, Liu Yang

**Affiliations:** 1 Institute of Mechatronic Control and Automatic Technology, Harbin University of Science and Technology, Harbin 150000, China; 1820500032@stu.hrbust.edu.cn (J.X.); yangliuheu@hrbust.edu.cn (L.Y.); 2 Key Laboratory of Advanced Manufacturing and Intelligent Technology Ministry of Education, Harbin University of Science and Technology, Harbin 150000, China; 3 State Key Laboratory of Robotics and System, Harbin Institute of Technology, Harbin 150000, China; rwb@hit.edu.cn

**Keywords:** micromanipulation, electrochemistry, microcomponents

## Abstract

The technology of picking up microcomponents plays a decisive role in the assembly of complex systems in micro- and nanoscale. The traditional method of picking up microcomponents with a mechanical manipulation tool can easily cause irreversible damage to the object, and only one object can be manipulated at a time. Furthermore, it is difficult to release the object with this method, and the release location is not accurate. With the aim of solving the above problems, the present study proposes an electrochemistry-based method for picking up metal microcomponents. First, the effect of ambient relative humidity on pickup was analyzed, and the effect of current density and electrolyte concentration on the deposition was examined. Then, a force analysis in the process of manipulation was carried out. Through the analysis of influence factors, the ideal experimental parameters were obtained theoretically. Finally, a simulation was carried out with COMSOL Multiphysics based on the above analysis. Copper microwires with a diameter of 60 μm and lengths of 300, 500, and 700 μm were successfully picked up and released using a pipette with a nozzle diameter of 15 μm. Compared with the traditional method, this method is simple to manipulate. Furthermore, it has a high success rate, causes less damage to the object, and good releasing accuracy.

## 1. Introduction

The technology of micromanipulation [1,2] is key to micro- and nanomanufacturing. The development of pickup technology [3,4] in micro- and nanoscale is vital for progress in the field of microassembly [5,6]. In the traditional method of picking up microcomponents, a mechanical manipulation tool [7,8] is used to clamp or adsorb an object. Power et al. [9] presented a tethered, 3D, compliant grasper that could prove useful for the investigation of biological microstructures, such as alveoli, villi, or even individual cells. When the manipulating object is brittle material, it can easily be damaged in the process of manipulation. Sun et al. [10] proposed an active control method to overcome adhesion force using inertia force and compound vibration, and polystyrene spheres with diameters of 30–80 μm were successfully picked up with this method. Xu [11] presented the design and validation of a new, robust impedance control for a gripper that was driven by a piezoelectric stack actuator. The device was verified through experimental studies on a grasp–hold–release operation of a graphite microrod. El-Khoury’s group [12] developed applications for the “ice” technology, which uses the adhesive properties of ice to pick up tiny components. However, the temperature of ice is low, which may affect the shape of some precision components. Most of the objects used in the traditional methods have been cells [13,14] or spherical particles [15]. At the same time, the traditional manipulation tool is too large for carrying out complex assembly work.

**Figure 1:**
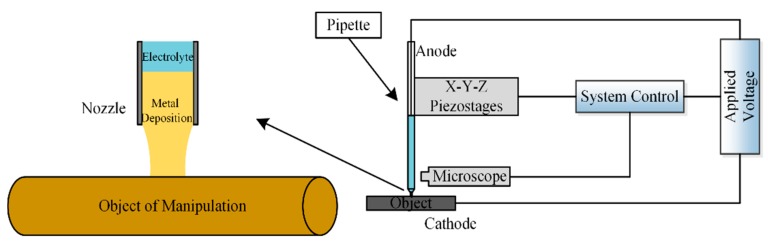
Diagram of the proposed manipulation method.

Kim et al. [16] investigated functional element coating on a plasma electrolytic oxidation (PEO)-treated Ti-6AL-4V alloy using the electrochemical precipitation method. However, there is limited research on the application of electrochemistry in micromanipulation. Based on the above research, this study proposes a method of picking up metal microcomponents using electrochemical deposition. As shown in Figure 1, this method replaces the traditional tool with a micropipette. During a pickup, an electrochemical reaction occurs, producing metal deposition. The metal deposition is a product of the electrochemical reaction, which is obtained by reducing copper ions on the cathode through the action of the current. The metal deposition and manipulating object are of the same metal element. The deposition connects the manipulating object with the micropipette, and the object can be picked up by manipulating the micropipette after sufficient deposition time.

## 2. Principles and Influencing Factors

### 2.1. Basic Principle and Experimental Device

**Figure 2:**
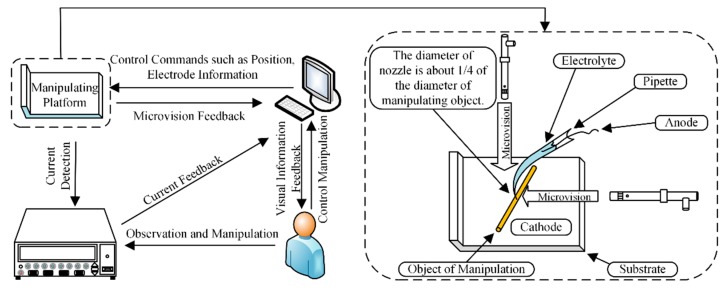
Experimental device and process of manipulation.

Figure 2 shows the experimental device and the process of manipulation. The pipette is connected to the micromotion platform, and the motion of the pipette is controlled by a computer. The deposition process is displayed on the computer in real time through a computer microvision. The diameter of the pipette is less than that of the manipulating object. This avoids the problem of the manipulating tool being too large to carry out complex pickup tasks. When carrying out an experiment, the micropipette filled with electrolyte is connected to the anode, the conductive substrate that carries the object is connected to the cathode, and the pipette is moved above the object. An external backside pressure can be applied to the pipette. The curvature of the electrolyte at the pipette front is concave, which prevents the electrolyte from touching the object, and the backside pressure allows the electrolyte in the pipette to form a protruding liquid front. The pipette is controlled to move downward so that the electrolyte touches the object to form a closed circuit. Electrochemical reaction can thus occur. The object is connected with the pipette through the deposition after sufficient deposition time. Controlling the pipette is therefore equivalent to controlling the object.

### 2.2. Effect of Ambient Relative Humidity on Pickup

The diffusion of water molecules [17,18] in a microenvironment is more obvious than that in a macroenvironment. In a microenvironment, when an electrolyte is going to touch an object, it contacts the air, and the water molecules of the electrolyte will be affected by the ambient relative humidity [19,20,21,22]. The higher the concentration gradient of water molecule in the environment and the electrolyte, the easier it is for the diffusion of the electrolyte water molecule. In order to reduce the effect of diffusion of water molecules on the quality of deposition, the distribution of ambient relative humidity was simulated.

**Figure 3:**
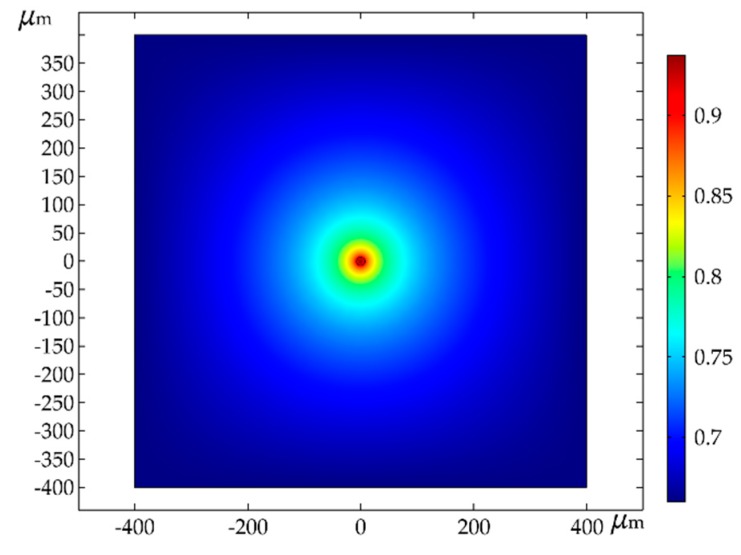
Relative humidity distribution of different areas.

During simulation, the nozzle diameter of the pipette was set to 15 μm. Ambient relative humidity was varied from 70% to 100% in the surrounding environment. The humidity along the nozzle opening was fixed at 100%, which accurately reflects the saturation condition at the water/air interface. From the nozzle to the surrounding environment, the relative humidity was reduced to a level that was close to actual environmental conditions. The simulation result is shown in Figure 3, which shows the distribution of relative humidity from the nozzle to the edge of the environment.

The result in Figure 3 shows that the relative humidity inside the nozzle was relatively stable, and the relative humidity of the external environment had a variance. When the diffusion of water molecules was stable, the relative humidity near the nozzle became 94%. The relative humidity of the nozzle edge decreased, indicating that the environment had a certain effect on the evaporation of water in the electrolyte. When the ambient humidity is low (30%–50%), the water molecules near the nozzle will diffuse due to the difference in the concentration gradient. The diffusion of water molecules results in the different concentration of water molecules inside the pipette and near the nozzle, which indirectly affects the rate of ion transport. Furthermore, excessive diffusion of water molecules may lead to the electrolyte separating out the crystal. This will indirectly affect the quality of the deposition. Combined with the above analysis, it is necessary to control the ambient relative humidity at about 94%.

### 2.3. Effect of Current Density on Pickup

**Figure 4:**
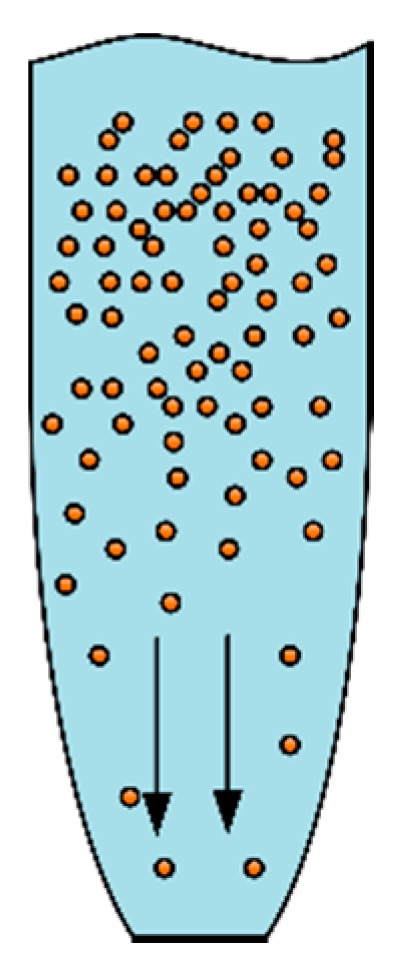
Schematic diagram of copper ion depletion near the edge of the nozzle.

In our experiment, the object was a copper microwire, and the electrolyte was a copper sulfate solution. In an electrochemical deposition, copper is obtained by reducing copper ions on the cathode through the action of the electric current. Current density is one of the important parameters of electrodeposition. The current density directly affects the deposition growth speed. The current density is expressed by Equation (1) [23]:

(1) $$J_{k}=\frac{I}{S}$$

where $J_{k}$ is the current density; *I* is the current passing through the pipette; and *S* is the cross-sectional area of the nozzle. The rate of metal deposition is proportional to the current passing through the pipette. Increasing current can accelerate metal deposition. However, over a certain range, copper ions in the electrolyte near the nozzle will be depleted due to the rapid deposition, which indirectly affects the deposition quality and growth. The schematic diagram of copper ion depletion is shown in Figure 4.

**Figure 5:**
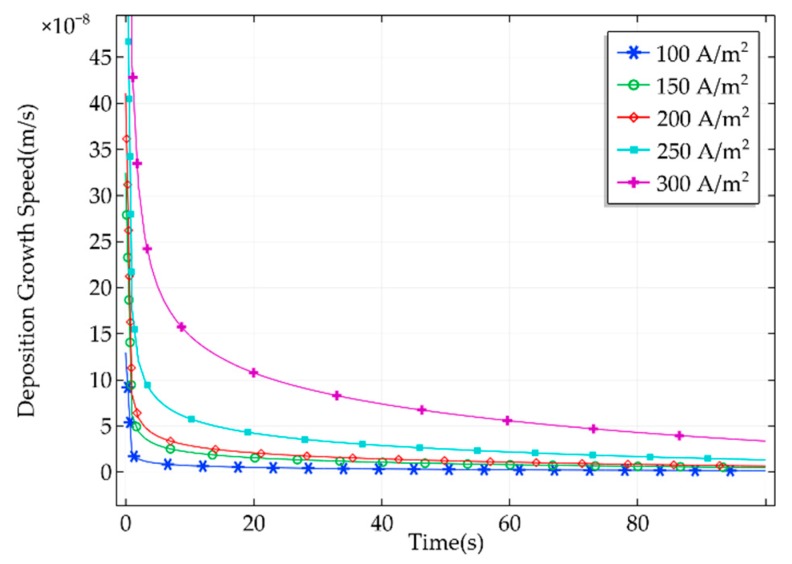
Deposition growth speed corresponding to different current densities.

In the deposition, the migration rate of copper ions from the inside of the pipette to the nozzle is the key factor affecting the deposition growth speed. Increasing current density will aggravate concentration polarization and affect the quality of the metal deposition. When the current density is too low, the electrochemical deposition time will increase, so the current density should not be too low. Combined with the above analysis, the current densities of 100, 150, 200, 250, and 300 $A/m^{2}$ were selected for the simulation. Deposition growth speed curves corresponding to different current densities are shown in Figure 5.

As can be seen from the curves in Figure 5, the deposition growth speed varied greatly at the start. With the passing of time, the deposition growth speed tended to be stable. The higher the current density, the greater the deposition growth speed. As a result, the faster the decrease in concentration of copper ions near the cathode, the greater the probability of copper ion depletion. Therefore, current density that is too high may affect the quality of metal deposition. The appropriate current density should be selected in the process of manipulation.

### 2.4. Effect of Electrolyte Concentration on Pickup

**Figure 6:**
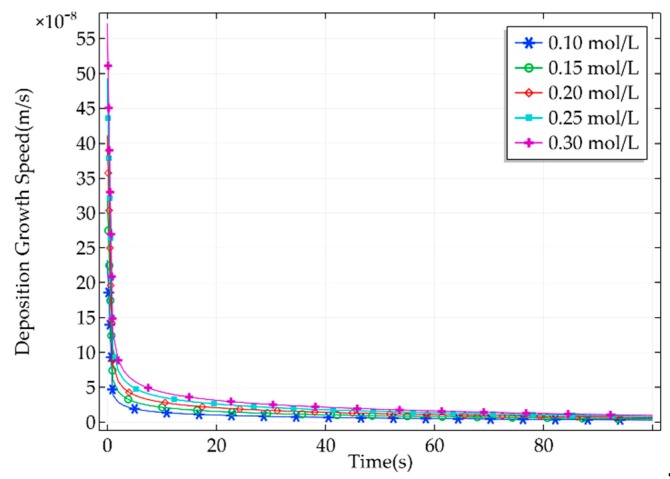
Deposition growth speed corresponding to different electrolyte concentrations.

Electrolytes with different concentrations have different effects on deposition growth speed in an electrochemical reaction. As the deposition proceeds, the concentration of copper ions in the electrolyte will decrease. Therefore, the effect of electrolytes with different concentrations on deposition was analyzed. A current density of 200 $A/m^{2}$ was chosen to simulate the effect of electrolyte concentration on the deposition. Copper sulfate solutions with concentrations of 0.10, 0.15, 0.20, 0.25, and 0.30 mol/L were selected for simulation. The deposition growth speed curves corresponding to different electrolyte concentrations are shown in Figure 6.

As can be seen from Figure 6, the deposition growth speed changed greatly at the start but then became stable after a short time. Within a certain range, increasing the electrolyte concentration accelerated the deposition speed. As can be seen, there were no extreme differences in the deposition growth speed with different electrolyte concentrations. When the electrolyte concentration is too high, concentration polarization may easily occur. This indirectly affects the deposition growth speed. When a low electrolyte concentration is selected for manipulation, the quality of the metal deposition can be easily affected by insufficient copper ion content. Therefore, the effect of copper ion content on metal deposition should be fully considered in the process of deposition, and appropriate electrolyte concentration should be selected for manipulation.

### 2.5. Effect of Force on Pickup

**Figure 7:**
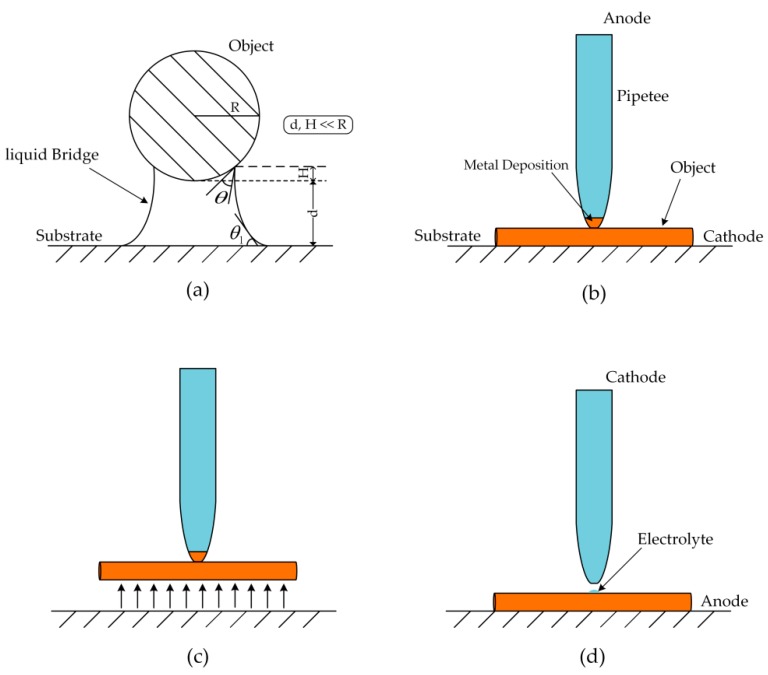
(**a**) The liquid bridge between the substrate and the copper microwire; (**b**) deposition; (**c**) pickup; (**d**) release.

During manipulation, the deposited copper cannot be broken by pulling. Therefore, the deposition needs to overcome the van der Waals force between the object and the substrate and the gravity of the object. Experiments and theory have demonstrated that, between 1 mm and 1 μm, when humidity increases (HR > 50%), a liquid bridge between two solid surfaces appears due to capillary force, increasing the reciprocal attraction [6]. During manipulation, the ambient humidity is quite high, resulting in a relatively high content of water molecules in the air. As a result, there is a liquid bridge between the substrate and the object (Figure 7). The metal deposition connects the pipette and the object, and the object is picked up by moving the pipette. At this time, the metal deposition must have enough strength to ensure that it will not be broken by pulling. As a result, the deposition needs to overcome the van der Waals force between the substrate and the object, the gravity of the object, and the capillary force. It is assumed that the deposition is cylindrical. The nozzle has enough strength to make sure it is not broken by pulling. The successful pickup needs to meet Equation (2):

(2) $$F_{VDW}\;+\;G_{Cu}\;+\;F_{c}\;<\;\sigma_{e\left(Cu\right)}\cdot S_{Nozzle}$$

where $F_{VDW}$ is the van der Waals force between the substrate and the copper microwire, which can be expressed by Equation (3); $G_{Cu}$ is the gravity of the copper microwire, which can be calculated with Equation (4); $F_{c}$ is capillary force between the substrate and the copper microwire, and the simplified capillary force can be calculated with Equation (5); $\sigma_{e\left(Cu\right)}$ is the tensile strength of the copper; and $S_{Nozzle}$ is the cross-sectional area of the nozzle. When the nozzle diameter is 15 μm, the maximum length of copper microwire with a diameter of 60 μm that can be picked up is 578 μm. Because the surface of the substrate and the object is not ideally smooth, the actual van der Waals force is smaller than the theoretical value. Therefore, the actual maximum length is larger than the theoretical value. Therefore, in the experiment, a copper microwire with a length of 700 μm could be picked up.

(3) $$F_{VDW}=\frac{A_{H}\sqrt{R}}{8\sqrt{2}\cdot d^{5/2}}\cdot L$$

where $A_{H}$ is the Hamaker constant; R is the radius of the copper microwire; d is the distance between the copper microwire and the substrate; and L is the length of the copper microwire.

(4) $$G_{Cu}=\rho_{Cu}\cdot \pi R^{2}\cdot L\cdot g$$

where $\rho_{Cu}$ is the density of copper, and g is the ratio of gravity to mass.

(5) $$F_{c}=\frac{(2\pi R+2L)\cdot \gamma_{1}\cdot \left(cos\theta +cos\theta_{1}\right)}{1+\frac{d}{h}}$$

where $F_{c}$ is the capillary force; h is the height of the liquid bridge infiltrating the copper microwire; $\gamma_{1}$ is the surface tension coefficient of water at room temperature; θ is the contact angle between the liquid bridge and the copper microwire; and $\theta_{1}$ is the contact angle between the liquid bridge and the substrate.

When releasing, the surface tension of the electrolyte between the copper microwire and the nozzle is as follows:

(6) $$F_{ST}=2\pi r_{Nozzle}\cdot \gamma_{2}\cdot \left(cos\theta_{2}+cos\theta_{3}\right)$$

where $F_{ST}$ is the surface tension; $\gamma_{2}$ is the surface tension coefficient of the copper sulfate solution at room temperature; $\theta_{2}$ is the contact angle between the copper microwire and the electrolyte; and $\theta_{3}$ is the contact angle between the nozzle and the electrolyte.

As long as the sum of the van der Waals force between the object and the substrate, the gravity of the object, and the capillary force between the object and the substrate is greater than the surface tension of the electrolyte, the object can be successfully released. After calculation, a pipette with a diameter of 15 μm was used to pick up the copper microwire with a diameter of 60 μm and a length of 300 μm, and it could be successfully released. In the experiment, copper microwires with lengths of 300, 500, and 700 μm were successfully picked up and released.

## 3. Simulation and Results

**Figure 8:**
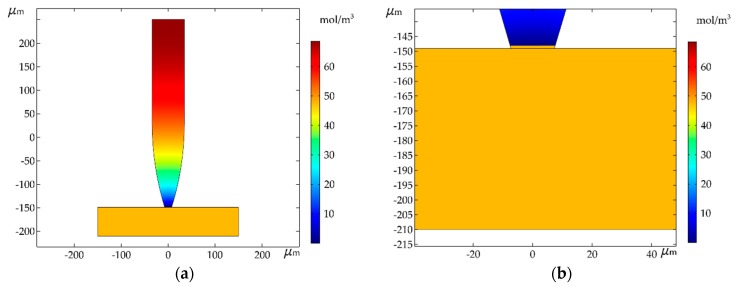
(**a**) Simulation results: distribution of copper ion concentration in the pipette after 100 s; (**b**) local enlargement of the simulation results.

After the above simulation and analysis, the ideal experimental parameters were finally obtained. Room temperature conditions can be easily achieved in practical manipulation, where the electrochemical reaction and molecular diffusion are relatively stable. In order to make the experimental results reliable and representative, the ideal parameters were selected for simulation. In the simulation, the electrolyte concentration was 0.20 mol/L, and the current density was 200 $A/m^{2}$. A pipette with a nozzle diameter of 15 μm was used to pick up copper microwires with a diameter of 60 μm and a length of 300 μm. The final simulation results are shown in Figure 8.

From Figure 8a, it can be seen that the deposition combined the manipulating object and the pipette as a whole. The object is shown as a horizontal rectangle in the figure, and the bottom of the pipette is the metal deposition. The pipette was connected with the object, and the manipulation of the object was realized by moving the pipette. Figure 8b is a partial enlargement of the deposition. It can be seen that the metal deposition was connected with the object to form a whole. When making the pipette, the shape of the nozzle was designed to be an inverted trapezoid, so the metal deposition during pickup was an inverted trapezoid. The diameter of the upper part of the deposition was larger than that of the lower part. Therefore, during the process of pickup, the metal deposition would not fall off from nozzle. The copper microwires can therefore be picked up by moving the pipette.

**Figure 9:**
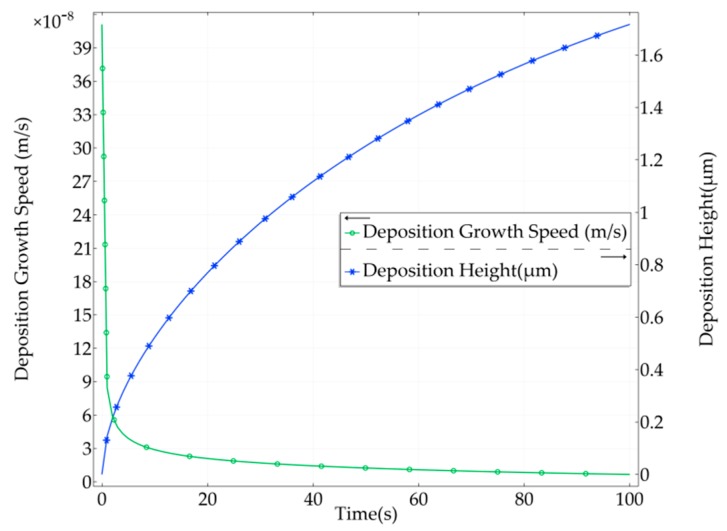
The relationship curves of deposition growth speed and height corresponding to time.

The relationship curves of the deposition growth speed and height corresponding to time are given in Figure 9. After 100 s, the deposition height was 1.7172 μm. Initially, the deposition height increased quickly with time. Subsequently, the change was more uniform. These results satisfy the relationship curve between deposition growth speed and time.

In this method, the object is moved to the designated position and the opposite voltage is applied to electrolyze the deposition, which transfers the deposition into the electrolyte. At this time, the metal deposition is equivalent to the anode. When the power supply is connected, an electrochemical reaction will take place, and the metal deposition obtained during pickup will be electrolyzed. When it is fully electrolyzed, the pipette and the object are connected only by the electrolyte. The diameter of the copper microwire is four times as large as the nozzle, so there is little liquid touching the object. Under the effect of van der Waals force between the object and the substrate, the gravity of the object, and the capillary force, the object can be successfully released by moving the pipette away from it. The metal deposition and the manipulating object are of the same metal element. The trace liquid left on the object during separation will evaporate quickly, so it will not pollute the object. During manipulation, the contact area between deposition and the object is quite small, which can minimize damage to the manipulating object.

After analysis, the ideal parameters were selected for simulation. The simulation results show that the deposition generated by the reaction combines the pipette with the object. The object can be picked up by moving the pipette. When picking up, the contact area between the deposition and the object is quite small, which can minimize damage to the manipulating object. This method can achieve a high manipulation success rate. At the same time, this method can be used to pick up objects of various shapes.

## 4. Conclusions

In this paper, an electrochemical method of picking up microcomponents is proposed. The effect of ambient relative humidity, current density, and electrolyte concentration were analyzed and simulated, and force analysis in the process of manipulation was carried out. The ideal experimental parameters were obtained theoretically. The simulation results showed that when the relative humidity was 94%, it had little effect on the diffusion of water molecules in the electrolyte. When the current density was 200 $A/m^{2}$ and the electrolyte concentration was 0.20 mol/L, the deposition quality was optimal. Combined with the above parameters, further simulation was carried out. Copper microwires with a diameter of 60 μm and lengths of 300, 500, and 700 μm were successfully picked up and released using a pipette with a nozzle diameter of 15 μm. Compared with the traditional mechanical method, this method has simple manipulation, high success rate, causes less damage to the object, and has good releasing accuracy. This method has potential in the field of microcomponent manipulation.
